# Proteomic Analysis of Retinal Tissue in an S100B Autoimmune Glaucoma Model

**DOI:** 10.3390/biology11010016

**Published:** 2021-12-23

**Authors:** Sabrina Reinehr, Annika Guntermann, Janine Theile, Lara Benning, Pia Grotegut, Sandra Kuehn, Bettina Serschnitzki, H. Burkhard Dick, Katrin Marcus, Stephanie C. Joachim, Caroline May

**Affiliations:** 1 Experimental Eye Research Institute, University Eye Hospital, Ruhr-University Bochum, In der Schornau 23-25, 44892 Bochum, Germany; sabrina.reinehr@rub.de (S.R.); janine.theile@rub.de (J.T.); Lara.Benning@uni-wh.de (L.B.); pia.grotegut@rub.de (P.G.); sandra.kuehn@rub.de (S.K.); Burkhard.dick@kk-bochum.de (H.B.D.); 2 Department Functional Proteomics, Medizinisches Proteom-Center, Ruhr-University Bochum, ProDi E2.227, Gesundheitscampus 4, 44780 Bochum, Germany; annika.guntermann@rub.de (A.G.); bettina.serschnitzki@rub.de (B.S.); katrin.marcus@rub.de (K.M.)

**Keywords:** autoimmune, normal-tension, proteomics, S100B, HSP60, α2-macroglobulin, DIA mass spectrometric analysis, glaucoma, mass spectrometry

## Abstract

**Simple Summary:**

Preventing blindness is an urgent need in a permanently further aging society. Glaucoma is one of the most common causes for blindness, but the exact pathomechanisms are not yet fully understood. Although an elevated intraocular pressure is a major risk factor, patients can have symptoms under normal pressure. Studies point towards an involvement of the immune system in glaucoma. Hence, in an animal model, where immunization with ocular antigens leads to intraocular-independent glaucomatous damage, we took a closer look into the pathophysiology with the help of proteomics. The proteomic analyses revealed significant alterations of proteins already at 7 and 14 days after immunization, before glaucomatous degeneration occurs. These proteins are often associated with the immune system. Hence, these data underline the important role of immunological factors in glaucoma. In the future, these factors might serve as disease markers.

**Abstract:**

Glaucoma is a neurodegenerative disease that leads to damage of retinal ganglion cells and the optic nerve. Patients display altered antibody profiles and increased antibody titer, e.g., against S100B. To identify the meaning of these antibodies, animals were immunized with S100B. Retinal ganglion cell loss, optic nerve degeneration, and increased glial cell activity were noted. Here, we aimed to gain more insights into the pathophysiology from a proteomic point of view. Hence, rats were immunized with S100B, while controls received sodium chloride. After 7 and 14 days, retinae were analyzed through mass spectrometry and immunohistology. Using data-independent acquisition-based mass spectrometry, we identified more than 1700 proteins on a high confidence level for both study groups, respectively. Of these 1700, 43 proteins were significantly altered in retinae after 7 days and 67 proteins revealed significant alterations at 14 days. For example, α2-macroglobulin was found significantly increased not only by mass spectrometry analysis, but also with immunohistological staining in S100B retinae at 7 and 14 days. All in all, the identified proteins are often associated with the immune system, such as heat shock protein 60. Once more, these data underline the important role of immunological factors in glaucoma pathogenesis.

## 1. Introduction

Glaucoma, one of the most common causes for blindness worldwide, comprises a variety of eye diseases, whose pathological hallmark is a progressive loss of retinal ganglion cells (RGC) and their corresponding axons [1]. In the initial disease stages, the core clinical feature is visual field loss, which often remains unnoticed by the patient. When patients are firstly clinical diagnosed, the neuropathy is unfortunately often far progressed due to a long asymptomatic clinical phase. Hence, about 10–50% of patients are unaware that they are affected by this disease [2,3,4,5,6]. The precise etiology in most people suffering from glaucoma is still unknown. High intraocular pressure (IOP) has been identified as the main risk factor, and it is known that blocking the axonal protein transport at the lamina cribrosa causes an initial axonal damage and RGC death by trophic insufficiency. However, normal-tension glaucoma (NTG) occurs in patients with physiological IOP [7] and accounts for about 30% of glaucoma cases [8].

In addition to elevated IOP, ischemic/hypoxic damage [9], astrocyte and glia cell alterations, as well as excessive stimulation of the glutamatergic system [10] are discussed as possible pathomechanisms. An involvement of the immune system is also considered [11,12,13], due to the observation of up- and down-regulations in the systemic as well as the ocular antibody profiles in glaucoma patients [14,15,16]. Additionally, antibody deposits were demonstrated in glaucomatous retinae [17]. It is likely that a combination of several pathogenic factors and mechanisms increases the possibility of developing glaucoma.

Currently, glaucoma treatment is based on sustained IOP lowering, which can slow down, but not halt, disease progression [18,19]. In addition, the side effects of the topical drug medication are not insignificant and can lead to ocular irritation, decreasing the compliance of patients [20]. The social, economic, and emotional burden that the blindness, resulting from the disease, poses on patients and their relatives should not be neglected. These facts emphasize the importance of discovering new pharmacological strategies to prevent patients from going blind and, with that, losing their autotomy.

For the investigation of pathomechanisms and novel therapies, it is necessary to have suitable models that allow for such screening. To inquire whether antibodies detected in glaucoma patients are part of glaucoma pathogenesis or a result of disease progression, the experimental autoimmune glaucoma (EAG) animal model was established. This animal model is based on findings in glaucoma patients. A high autoantibody titer against S100B, a small calcium binding protein, was detected in samples from glaucoma patients [21]. In the central nervous system, S100B is mainly expressed by glial cells, such as oligodendrocytes, Schwann cells, ependymal cells, retinal Müller cells, and astrocytes [22]. S100B regulates and maintains the homeostasis of the important second messenger calcium and is, therefore, involved in many cell activities, such as signal transduction, cell differentiation, the regulation of cell motility, transcription, and cell cycle processes [23,24]. Extracellularly, S100B can act as a signal molecule and bind to receptors, such as the receptor for advanced glycation end products (RAGE). In high concentrations, S100B can have negative effects and lead to cell death. For example, the binding of RAGE can induce the activation of microglia cells, leading to a release of proinflammatory cytokines to an excessive extent [25]. Furthermore, there seems to be a link between S100B and different neuronal diseases [26].

In a previous study, we could observe that immunization with S100B in rats led to a significant loss of RGCs after 28 days and a fast degeneration of optic nerves. Interestingly, the IOP in this model was not altered [27]. Therefore, S100B immunization can be used to mimic the effects seen in NTG. The purpose of the presented study was to discover pathological pathways underlying glaucoma using the EAG model. Therefore, retinae were prepared and lysed; then, altered proteins were, for the first time, analyzed with mass spectrometry in data-independent acquisition (DIA) mode 7 and 14 days after S100B immunization. DIA leads, in contrast to data-dependent acquisition, to a more complete map of the fragment ion spectra, because all ions in a predefined mass/charge (*m*/*z*) range (or, time window) are fragmented. Based on this, DIA is gaining increasing importance for comprehensive label-free mass spectrometric analysis. Additionally, we performed immunohistological evaluations of different proteins, which were found to be altered in the mass spectrometry study.

## 2. Materials and Methods

### 2.1. Animals

All procedures concerning animals adhered to the ARVO statement for the use of animals in ophthalmic and vision research. All experiments involving animals were approved by the animal care committee of North Rhine–Westphalia, Germany, and were performed in accordance with relevant guidelines and regulations (approval codes: 84-02.04.2013_A291 and 81-02.04.2019_A071).

Male Lewis rats (Charles River, Wilmington, MA, USA), 6 weeks of age, were used for the experiments and kept under environmentally controlled conditions with free access to chow and water (*n* = 9/group/point in time). Detailed observations and health checks, including eye exams, were performed regularly, as described previously [28].

### 2.2. Immunization

Rats received a single dose of 1 mg/mL S100B (Sigma–Aldrich, St. Louis, MO, USA) intraperitoneally [29]. The antigen was first mixed with incomplete Freund’s adjuvant (200 µL) plus 3 µg pertussis toxin (both Sigma–Aldrich). The animals of the control group were injected with 0.9% sodium chloride with equivalent doses of Freund’s adjuvant and pertussis toxin.

To obtain the retinae for proteomic analysis and (immune-)histology, animals were sacrificed 7 and 14 days after immunization by carbon dioxide inhalation.

### 2.3. Mass Spectrometric Analysis of Retinae Samples

The preparation of 6 pooled retinae samples for the creation of a retina-specific spectral library was described previously by Reinehr et al. [30]. Briefly, the eyes were enucleated, retinae were carefully extracted, and homogenization was performed by sonication.

The preparation of the actual study samples (*n* = 8/group/point in time) for DIA-based measurements was performed with minor changes. In total, 20 µg of each retina lysate was loaded on a NuPAGE™ 10% Bis–Tris gel (Fisher Scientific Inc., Waltham, MA, USA). Gel electrophoresis was limited to 50 V for 15 min. After stopping electrophoresis, gels were stained with Coomassie blue (SimpleBlue™ SafeStain, Fisher Scientific Inc.) according to the manufacturer’s instructions. The resulting single protein band per lane was dissected and transferred into a new glass vial. The protein bands were destained, pH was adjusted, and disulphide bridges were reduced as well as modified. After finishing the incubation cycle, gel pieces were dried and resuspended in 9.5 µL trypsin solution (0.033 µg/µL; SERVA Electrophoresis GmbH, Heidelberg, Germany) plus 12 µL 50 mM ammonium bicarbonate. Trypsin digestion and the elution of peptides were performed as described earlier [30]. The resulting peptide extract was completely dried in a vacuum concentrator and resuspended in 30 µL 0.1% (*v*/*v*) trifluoroacetic acid. The peptide concentration was determined with amino acid analysis [31]. For mass spectrometric analysis, 80 ng of this peptide extract were transferred to a new glass vial and filed up to a volume of 14.5 µL with 0.1% (*v*/*v*) trifluoroacetic acid. Finally, 1 µL “indexed Retention Time” (iRT) peptides (Biognosys AG; Schlieren, Switzerland) were added to each retina sample.

The mass spectrometric analysis for the study samples was comparable to the spectral library samples performed with a Q Exactive™ HF mass spectrometer (Fisher Scientific Inc.) but in DIA mode. The scan range for the full MS1 was set to 350 to 1100 *m*/*z* with a resolution of 120,000. Fragmentation was performed by HCD with a resolution of 30,000 and a stepwise NCE of 25.5%, 27%, and 30%. The first fixed mass was set to 200 *m*/*z* (ACG 3e6, maximum injection time automatic) and the default charge state set to ≥+4. The thereby generated date set for the study samples has been uploaded to ProteomeXchange with the identifier PXD023995.

### 2.4. Data Evaluation and Functional Analysis

Acquired mass spectrometric data were analyzed with the interface Spectronaut™ Pulsar (Biognosys) with default settings and minor changes, as used in Barkovits et al. [32]. Briefly summarized, the retina sample dataset described previously [30] was taken to generate a retina-specific spectral library (reference data set). The false discovery rate (called Qvalue) was set to a threshold of 1%. For further statistical evaluation, proteins had to be quantified in at least 80% of the samples of one study group. Further filter criteria, such as a ratio >30% (fold change of more than 1.3) and a Student’s *t*-test with *p* < 0.05, were calculated manually (* *p* < 0.05, ** *p* < 0.01, *** *p* < 0.001).

For a functional proteomic analysis, the Reactome pathway database was used, a curated database for the visualization, interpretation, and analysis of pathway knowledge [33]. Importantly, all protein IDs were mapped to human equivalents prior to analysis. A binomial test was used to calculate the probability shown for each result and the *p*-values were corrected for the multiple testing (Benjamini–Hochberg procedure) that arises from evaluating the submitted list of identifiers against every pathway. Additionally, all proteins were further evaluated with the STRING bioinformatic database, which provides a comprehensive overview of the direct and indirect relationships as well as the interactions between proteins [34].

### 2.5. Tissue Preparation for Immunohistology

At 7 and 14 days after immunization, the control and the S100B eyes were enucleated and fixed in 4% paraformaldehyde for 60 min. Subsequently, the rat eyes were treated with 30% sucrose and were embedded in a Neg-50 compound (Tissue Tek; Fisher Scientific Inc.). On a cryostat (Fisher Scientific Inc.), 10-µm cross-sections were cut, mounted on microtome slides (Histobond, Paul Marienfeld GmbH & Co. KG, Lauda-Königshofen, Germany), and dried overnight. Afterwards, the sections were fixed in ice-cold acetone (VWR, Radnor, DE, USA) for 10 min.

### 2.6. Hematoxylin and Eosin Staining

Exemplary retinal cross-sections from both groups and points in time were stained with hematoxylin and eosin (H&E; both Merck, Darmstadt, Germany), dehydrated in ethanol, and treated with xylene before the sections were covered with Eukitt (O.Kindler, Bobingen, Germany). Images were acquired at 200× magnification using an Axio Imager M1 microscope (Zeiss, Oberkochen, Germany).

### 2.7. Immunofluorescence Staining

The immunofluorescence staining of specific cell types in the retina was performed as described previously (*n* = 5/group/point in time) [27]. First, all cross-sections (6 sections/eye) were blocked with a mixture of 10–20% serum, 0.1% TritonX-100 (Sigma–Aldrich), and PBS (Biochrome, Schaffhausen, Germany) for one hour. The specific first antibodies (Table S1) were diluted in the same mixture and incubated overnight at room temperature. After three washing steps with PBS, all sections were incubated with Alexa Fluor 555- or Alexa Fluor 488-labelled secondary antibodies (Table S1). To visualize cell nuclei, 4′,6 diamidino-2-phenylindole (DAPI; SERVA Electrophoresis GmbH), diluted in distilled water, was applied to the sections. Finally, the sections were covered with Shandon-Mount (Fisher Scientific Inc.). Negative controls were performed for all antibody stains using only secondary antibodies.

### 2.8. Histological Evaluation

Four images (two peripheral and two central) per retinal cross-section were acquired at 400× magnification using the Axio Imager M2 microscope (Zeiss). Afterwards, an equal area of each picture was cut out using Corel Paint Shop software (Corel Corporation, Ottawa, ON, Canada).

The number of RNA-binding proteins with multiple splicing (RBPMS)^+^ cells in the ganglion cell layer and calretinin^+^ cells in the inner nuclear layer were counted using ImageJ software (National Institute of Health, Bethesda, MD, USA). To measure the area of HSP60 and α2-macroglobulin, pictures were processed using an ImageJ macro (National Institute of Health), as described previously [35,36]. Briefly, pictures were converted into greyscale (32-bit), and a rolling ball radius of 40 pixels was subtracted to minimize background interference. Further, a proper lower threshold was determined for each picture, which was achieved when the greyscale picture corresponded to the original one. At the end, the mean value was calculated, and this number was used for the final analyses (HSP60: 15.49; α2-macroglobulin: 10.36). The upper threshold was set as the highest number out of all pictures (HSP60: 254.90; α2-macroglobulin: 246.01). Between these defined lower and upper thresholds, the percentage of the labeled area of the staining was measured.

### 2.9. Statistics of Immunohistology Evaluations

Immunohistology data were shown as mean ± standard error of the mean (SEM) and were statistically analyzed using the Statistica software (V13.3; Dell, Round Rock, TX, USA). Values of the control groups were set to 100%. The S100B group was compared to the controls by applying Student’s *t*-test with *p* < 0.05 considered statistically significant. * *p* < 0.05, ** *p* < 0.01, and *** *p* < 0.001.

## 3. Results

**Figure 1:**
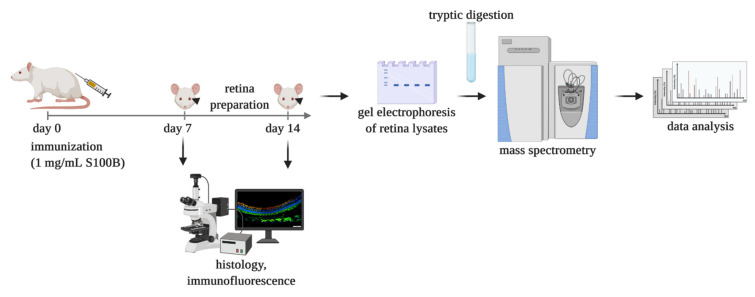
Study overview. Schematic study setup created with biorender.com (accessed on 15 October 2021).

The study compromises two study groups, the S100B and the control group, with, in total, 18 rats per group for proteomic analysis. Respectively, nine rats from each study group were prepared 7 days after immunization and the remaining nine rats per group 14 days after, for proteomic analysis. One sample from each group has been excluded due to insufficient protein yield after tissue preparation. Additionally, five retinae/group/point-in time were prepared for (immuno-)histology (Figure 1).

### 3.1. Characteristics of Proteins in the Retina-Specific Spectral Library

For the characterization of differences within the proteome in consequence of immunization with S100B, a DIA-based mass spectrometry analysis was chosen. To this end, the before-generated retina-specific spectral library served as a consensus template [30]. It consists of mass spectrometric data of the ocular sinister of six rats acquired by data-dependent acquisition mass spectrometry. In this generated spectral library, 67,165 peptides, which were assigned to 4689 proteins, have been identified. For a more detailed characterization of these proteins, a pathway enrichment analysis was carried out using the Reactome pathway database [33]. As a result, one of at least 2725 mapped proteins covered 1988 biological pathways (Figure S1).

### 3.2. Comparative Proteomic Analysis Revealed Complex, Time-Dependent Regulations after S100B Immunization

**Figure 2:**
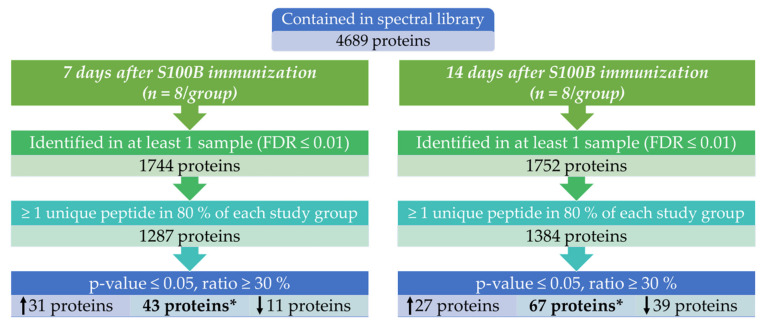
Flow chart of identified and filtered proteins for study groups at 7 as well as 14 days after S100B immunization (*n* = 8/group). * One protein showed both an up-regulated as well as a down-regulated peptide, compared to the control group.

Based on the spectral library, the actual retinal proteomic data analysis in the framework of S100B immunization was carried out. The filtering steps during data evaluation are displayed in Figure 2. Within all 7-day study samples (*n* = 8/group), 13,199 peptides, assigned to 1744 proteins, were identified. Proteins identified with at least one unique peptide in at least 80% of the control as well as the S100B samples were selected for further comparative proteome analysis. These 1287 proteins are reported in Table S2. Comparing the abundancies of the 7-day study groups, 43 proteins had a ratio of at least > 30%, a *p*-value < 0.05, and a FDR < 0.01. Of these, 11 proteins were under-represented and 31 over-represented in the S100B study group 7 days after immunization (Figure 2).

Interestingly, stress-induced-phosphoprotein 1 revealed one peptide that was over-represented, while another peptide assigned to this protein was under-represented in the S100B study group. The abundance of the 20 most differential proteins, when focusing on the smallest *p*-value 7 days after S100B immunization, is presented in Table 1.

**Figure 3:**
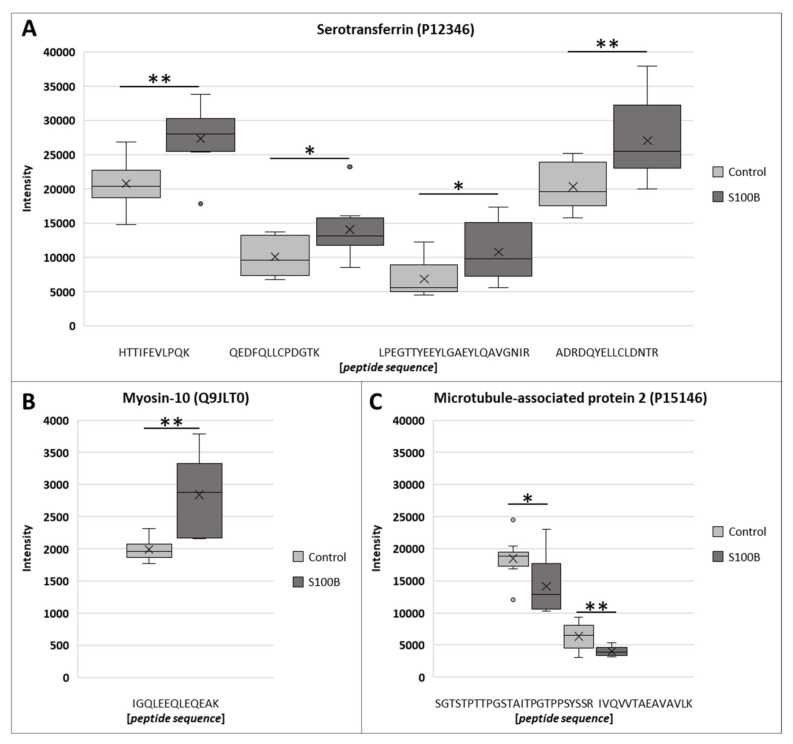
Mass spectrometry-based intensities of differential peptides with their assigned proteins 7 days after S100B injection. (**A**) Four unique peptides associated with serotransferrin and (**B**) one unique myosin-10 peptide were significantly up-regulated in rat retinae, 7 days after S100B immunization. (**C**) In contrast, two unique peptides of microtubule-associated protein 2 were significantly down-regulated in in rat retinae, 7 days after S100B immunization (* *p* < 0.05, ** *p* ≤ 0.01, ratio > 30%).

The results of three exemplary differential proteins, known from the glaucoma literature, are visualized in Figure 3. Serotransferrin had four, myosin-10 had one, and microtubule-associated protein 2 (MAP-2) had two unique peptides, which were significantly expressed based on the filtering criteria.

The study group data for the 14-day time point were analogously evaluated and revealed 14,627 peptides that could be assigned to 1752 proteins. A total of 1384 proteins had at least one unique peptide and were identified in at least 80% of each study group (Table S3). Analysis of the abundancies of the 14-day time point study groups revealed 67 proteins with a ratio of at least > 30%, a *p*-value < 0.05, and a FDR < 0.01. Over-represented in the S100B study group were 27 proteins, under-represented were 39 proteins (Figure 2). Additionally, for this time point, cytoplasmic dynein 1 heavy chain 1 was a protein with an over-represented as well as an under-represented peptide in the S100B study group. The 20 most differential proteins 14 days after S100B injection with the smallest *p*-values are listed in Table 2.

**Figure 4:**
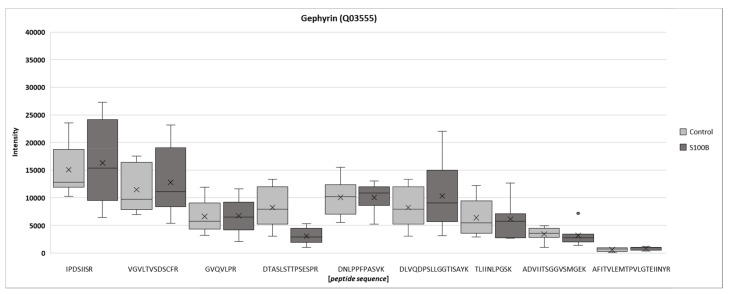
Mass spectrometry-based intensities of gephyrin 14 days after S100B injection. Nine unique peptides associated with gephyrin were differentially expressed in rat retinae 14 days after S100B immunization compared to control retinae (no significance).

In Figure 4, all identified peptides belonging to the protein gephyrin are shown. Recently, a significantly lower inhibitory post-synapse signal was visualized by anti-gephyrin in a later stage of retinal degeneration. Here, the intensity remained unchanged in comparison to the control group, confirming a recent study result - that 14 days after immunization might be too early in the retinal degeneration process of systemically immunized S100B animals [35].

In total, 58 proteins were over-represented and 50 proteins under-represented, in the comparative analysis at both points in time after the S100B immunization.

### 3.3. Characteristics of Differential Proteins

For a more biological characterization of the identified proteins present in the rat retina after S100B immunization, they were analyzed with the Reactome pathway database. The results of the Reactome pathway analysis of the proteins contained in rat retinae after S100B immunization are visualized in Figure S2. For the retina samples of the 7-day immunization, 1225 proteins were mapped to 1641 biological pathways in total. Strong pathways were, e.g., found in the area metabolism of carbohydrates. In comparison, 1637 biological pathways were associated with at least one of the 1234 retinal proteins from S100B immunization after 14 days. Thereby, a similar pathway distribution compared to the other group was detectable. However, there seems to be a stronger over-representation of some proteins 14 days after S100B immunization.

In a next step, the STRING database provided a detailed protein–protein network of the 43 significant proteins (FDR < 0.01; *p* < 0.05; ratio > 30%) 7 days after S100B immunization (Figure S3). There were mainly functional interactions related to the citric acid cycle, confirming even more that at least some individuals with glaucoma may have an impaired RGC energy metabolism [37]. In contrast, 14 days after S100B immunization, 67 significant proteins (FDR < 0.01; *p* < 0.05; ratio > 30%) formed interaction networks that, among others, showed an involvement in glutamate processing (Figure S4). This indicates the controversial role of glutamate in retinal excitotoxicity and neuroprotection [38,39].

### 3.4. No Loss of Retinal Ganglion Cells

**Figure 5:**
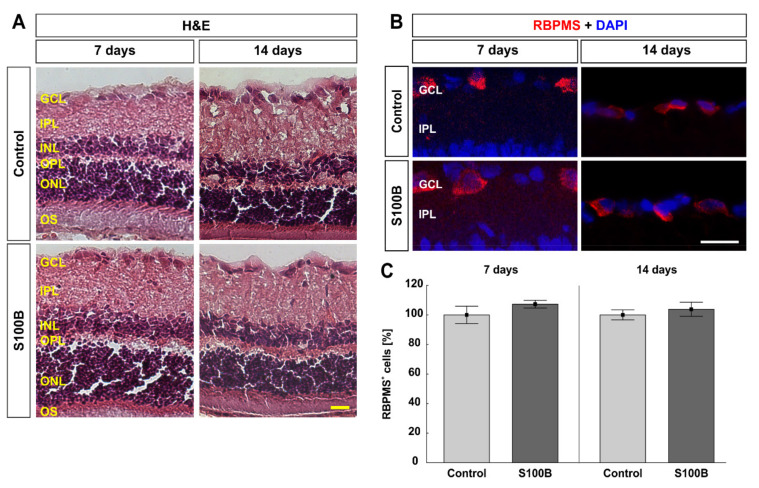
Intact retinal morphology and no retinal ganglion cell loss. (**A**) At 7 and 14 days after S100B immunization, the retinae showed no signs of inflammation or damage of the retinal layers. The morphology was comparable to those of the control group. (**B**) At 7 and 14 days after immunization, retinal cross-sections were labeled with an antibody against RBPMS (red; retinal ganglion cells). DAPI was used for counterstaining cell nuclei (blue). (**C**) The number of RBPMS^+^ cells remained comparable between S100B and control animals at 7 and 14 days after immunization. Values are mean ± SEM and control values were set to 100%. GCL = ganglion cell layer; IPL = inner plexiform layer; INL = inner nuclear layer; OPL = outer plexiform layer; ONL = outer nuclear layer; OS = outer segment. Scale bars: 20 µm.

Exemplary retinal cross-sections were stained with H&E at 7 and 14 days after immunization (Figure 5A). After S100B immunization, retinal layers appear normal in morphology, with no signs of inflammation or infiltration, compared to the controls at both points in time.

At 7 and 14 days after immunization, the number of RGCs was assessed by labeling retinal cross-sections with an antibody against RBPMS (Figure 5B). At 7 days, the number of RGCs was comparable in S100B (107.26 ± 2.57%) and control retinae (100.00 ± 5.89%; *p* = 0.292; Figure 5C). The number of RBPMS^+^ RGCs was still not altered between the S100B (103.80 ± 4.77%) and control animals (100.00 ± 3.33%; *p* = 0.532; Figure 5C) after 14 days. A previous study revealed a RGC loss later on in this model, 28 days after S100B immunization [27].

### 3.5. Altered Expression of Calretinin^+^ Amacrine Cells

**Figure 6:**
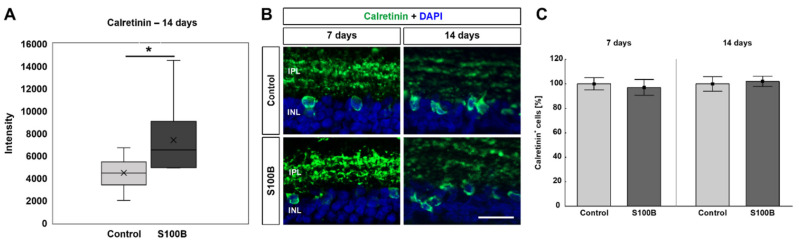
Altered calretinin expression. (**A**) Mass spectrometry analyses showed a significant up-regulation of the calretinin intensity (peptide sequence: GFLSDLLK) 14 days after immunization (*p* = 0.023). (**B**) Retinal cross-sections were stained with an anti-calretinin antibody (green) 7 and 14 days after immunization. DAPI was applied to visualize cell nuclei (blue). (**C**) The number of calretinin^+^ cells was not altered at either point in time. Values for immunohistology are mean ± SEM and control values were set to 100%. IPL = inner plexiform layer; INL = inner nuclear layer. Scale bar: 20 µm. * *p* < 0.05.

Mass spectrometry-based intensities of calretinin (peptide sequence: GFLSDLLK) revealed a significant up-regulation in S100B retinae 14 days after immunization (*p* = 0.023; Figure 6A). Then, possible changes in the number of amacrine cells were elaborated through an anti-calretinin staining 7 and 14 days after immunization (Figure 6B). At 7 days, the number of calretinin^+^ cells was comparable in the S100B (97.04 ± 6.47%) and control retinae (100.00 ± 4.93%; *p* = 0.364; Figure 6C). Further, no alterations were noted in the number of calretinin^+^ amacrine cells in the S100B retinae (102.06 ± 4.20) compared to the controls (100.00 ± 5.98%; *p* = 0.785) 14 days after immunization (Figure 6C).

### 3.6. Different HSP60 Expression after Immunization

**Figure 7:**
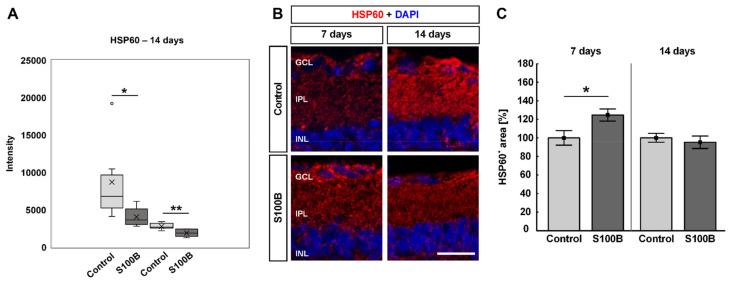
Altered HSP60 expression. (**A**) At 14 days after S100B immunization, the intensity of two peptide sequences of HSP60 was significantly down-regulated (peptide sequences: KPLVIIAEDVDGEALSTLVLNR: *p* = 0.033; DMAIATGGAVFGEEGLNLNLEDVQAHDLGK: *p* = 0.004). (**B**) An anti-HSP60 antibody (red) was applied on retinal cross-sections 7 and 14 days after immunization. DAPI was used to stain cell nuclei (blue). (**C**) At 7 days, the HSP60^+^ staining area was significantly increased in S100B animals (*p* = 0.042). No alterations were revealed between S100B and control retinae after 14 days. Values for immunohistology are mean ± SEM and control values were set to 100%. GCL = ganglion cell layer; IPL = inner plexiform layer; INL = inner nuclear layer. Scale bar: 20 µm. * *p* < 0.05; ** *p* < 0.01.

Retina samples, which were analyzed using mass spectrometry, showed that the intensity of two peptide sequences of HSP60 (peptide sequences: KPLVIIAEDVDGEALSTLVLNR: *p* = 0.033; DMAIATGGAVFGEEGLNLNLEDV-QAH-DLGK: *p* = 0.004) were significantly down-regulated in S100B animals compared to controls (Figure 7A). The staining area of HSP60 was evaluated by immunohistology 7 and 14 days after immunization (Figure 7B). After 7 days, a significant increase of the HSP60^+^ staining area was revealed in S100B rats (124.76 ± 6.57%) compared to controls (100.00 ± 7.89%; *p* = 0.042; Figure 7C). The percentage of the HSP60^+^ area in S100B animals (95.32 ± 6.71%) went back to control level (100.00 ± 4.73%; *p* = 0.584) after 14 days (Figure 7C).

### 3.7. Increased Levels of α2-Macroglobulin in S100B Retinae

**Figure 8:**
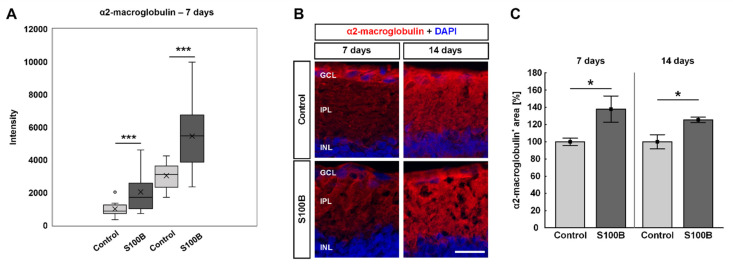
More α2-macroglobulin expression after immunization. (**A**) At 7 days after S100B immunization, the intensities of two peptide sequences of α2-macroglobulin were significantly up-regulated (both: *p* = 0.006). (**B**) Retinal cross-sections were labelled with an antibody against α2-macroglobulin (red), while DAPI counterstained cell nuclei (blue). (**C**) The staining area of α2-macroglobulin was significantly increased 7 (*p* = 0.044) and 14 days after immunization (*p* = 0.020). Values for immunohistology are mean ± SEM and control values were set to 100%. GCL = ganglion cell layer; IPL = inner plexiform layer; INL = inner nuclear layer. Scale bar: 20 µm. * *p* < 0.05; *** *p* < 0.001.

Mass spectrometric analysis of α2-macroglobulin revealed an up-regulation of two different peptide sequences (peptide sequences: KPLVIIAEDVDGEALSTLVLNR and DMAIATGGAVFGEEGLNLNLEDVQAHDLGK) in S100B animals compared to controls 7 days after immunization (both: *p* = 0.006; Figure 8A). At 7 and 14 days after immunization, retinal cross-sections were labelled against anti-α2-macroglobulin (Figure 8B). A significantly larger α2-macroglobulin^+^ staining area could be noted in S100B retinae (137.95 ± 15.31%) compared to control ones (100.00 ± 4.19%; *p* = 0.044) after 7 days (Figure 8C). Further, the α2-macroglobulin^+^ staining area was significantly increased in S100B animals (125.53 ± 3.16%) when compared to control retinae (100.00 ± 8.21%; *p* = 0.020) 14 days after immunization (Figure 8C).

### 3.8. Up-Regulation of Interphotoreceptor Matrix Proteoglycans and Plastin 3

**Figure 9:**
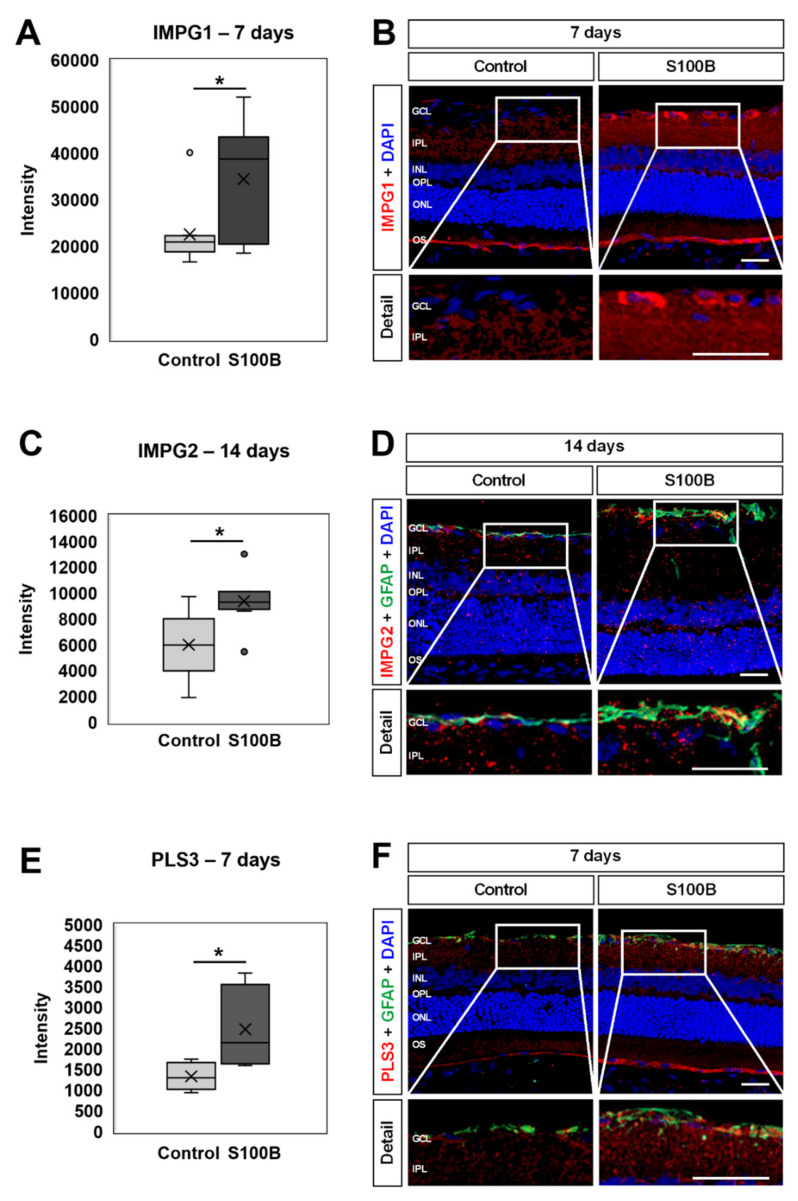
Up-regulated expression of various matrix proteins. (**A**) Mass spectrometry revealed an up-regulation of IMPG1 in S100B rats 7 days after immunization (*p* = 0.042). (**B**) Representative staining of anti-IMPG1 (red) on retinal cross-sections at 7 days. DAPI (blue) counterstained cell nuclei. IMPG1 staining was more distinct in S100B animals with the signal predominantly in the ganglion cell layer (GCL; detail image), the inner nuclear layer, and the outer segment. (**C**) A significant up-regulation of IMPG2 was observed in S100B animals 14 days after immunization (*p* = 0.031). (**D**) At 14 days, retinal cross-sections were labeled with an antibody against IMPG2 (red) and anti-GFAP (astrocytes; green), while DAPI stained cell nuclei (blue). In S100B retinae, IMPG2 signal was predominantly located in the GCL (detail image) and often co-localized with GFAP. (**E**) Analysis using mass spectrometry showed a significant up-regulation of PLS3 in S100B rats 7 days after immunization (*p* = 0.012). (**F**) At 7 days, retinae were labeled with antibodies against PLS3 (red) and GFAP (green). Cell nuclei were counterstained with DAPI (blue). PLS3 staining was more distinct in S100B animals. PLS3^+^ signal was localized in the GCL (detail image), inner nuclear layer, and outer segment. In the GCL, PLS3 was often co-localized with GFAP. GCL = ganglion cell layer; IPL = inner plexiform layer; INL = inner nuclear layer; OPL = outer plexiform layer; ONL = outer nuclear layer; OS = outer segment. Scale bars: 20 µm, scale bars detail: 10 µm. * *p* < 0.05.

Further interesting proteins were found up-regulated by mass spectrometric analysis. The photoreceptor-specific extracellular matrix protein (ECM) interphotoreceptor matrix proteoglycan (IMPG) 1, for example, was significantly increased in S100B retinae 7 days after immunization (*p* = 0.042; Figure 9A). Immunohistological staining showed a distribution in control animals predominantly in the outer segment, while in S100B rats, IMPG1^+^ staining could also be revealed in the ganglion cell layer (GCL; Figure 9B).

In addition, IMPG2 was found up-regulated in S100B rats compared to controls 14 days after immunization (*p* = 0.031; Figure 9C). Here, the distribution of the IMPG2 staining was not that evident in controls. In S00B retinae, IMPG2 staining was especially observed in the GCL, often co-localized with the astrocyte marker GFAP (Figure 9D).

At 7 days after S100B immunization, the actin-binding and bundling protein plastin 3 (PLS3) was found up-regulated (*p* = 0.012; Figure 9E). Immunohistological staining showed a weak distribution throughout the whole retina, with more intense signal in the outer segment in control rats. In S100B retinae, a stronger PLS3 staining, especially in the GCL and inner plexiform layers, could be revealed. In the GCL, the PLS3 staining was often co-localized with GFAP (Figure 9F).

## 4. Discussion

To identify key proteins which might play a role in the pathogenesis of glaucoma independent from elevated IOP, an established autoimmune glaucoma model was used for a proteomic analysis of retinae in this study. We previously demonstrated that an immunization with S100B leads to loss of RGCs and optic nerve degeneration after 28 days [27]. Nonetheless, (immunological) factors contributing to cell death are often found before a significant degeneration occurs. For example, a higher number of complement factors and more NFκB^+^ cells were revealed 7 and/or 14 days after S100B immunization [40]. Hence, to determine possible early disease markers for glaucoma, we analyzed rat retinae 7 and 14 days after immunization. At these points in time, the number of RGCs was not altered between S100B and control animals, which is in accordance with previous findings [41]. This slow progression of cell loss mimics the damage course in glaucoma-like events [42].

We identified several altered proteins after S100B immunization in the current study. We chose to take a closer look at different proteins that are either already connected to glaucomatous neurodegeneration, e.g., HSP60, or that were of interest but were not previously linked to glaucoma, e.g., IMPG1.

In the brain, MAP-2 is the most abundant MAP. Microtubules are one of the major components of the neuronal cytoskeleton [43]. In our study, we found a down-regulation of two unique peptides of MAP-2 7 days after immunization. In the mammalian retina, MAP-2 expression is reported especially in RGCs, amacrine cells, and the inner segments of photoreceptors [44,45,46]. An up-regulation of MAP-2 is associated with stabilizing dendrites [47]. Hence, a down-regulation could hint towards a destabilization and could be a first sign of dendrite and cell loss. In R28 neuroretinal cells, an internalization of the amyloid beta peptide led to a transient disruption of MAP-2 [48]. In donor retinae of patients with age-related macular degeneration, MAP-2 labelling was noted in the inner segments of abnormal photoreceptors with abnormally located nuclei. Since not all abnormal neurons were MAP-2 positive, Pow and Sullivan assumed that MAP-2 expression might be transient and only occur during neuro-morphogenesis [49].

In the mammalian retina, amacrine cells are the largest cohort of neurons [50,51,52]. Amacrine cells are interneurons, which provide the integration of signals that are essential to the construction of the RGC visual message sent to the brain [53]. Further, they are highly diverse and use several neurotransmitters. Based on their ramification in the inner plexiform layer, amacrine cells can be classified into different subtypes. Those who use glycine are so-called small-field amacrine cells and the most common ones are class AII cells. Calretinin is localized in different neurons in the retinae of vertebrates [54]. Specifically, in the rat retina, direction-selective cholinergic cells contain calretinin [55]. The mass spectrometric analysis in our study showed an up-regulation of calretinin 14 days after S100B immunization. In contrast, the number of calretinin^+^ cells in the inner nuclear layer counted after immunofluorescence staining remained unaltered. The discrepancy between those data can be explained by the different methods used. While, for immunohistology, only calretinin^+^ cells in the inner nuclear layer were counted, for mass spectrometry, the whole retina was processed and analyzed. Further, amacrine cells project presynaptic dendrites to the inner plexiform layer where they connect by synapses with RGCs [56]. Possibly, the increase of calretinin protein points towards early compensatory mechanisms, which were similarly described in Alzheimer’s disease. Here, at first, the dysfunction of synapses and neurons triggers a compensatory response to maintain synaptic connectivity. Therefore, new synapses are formed and, in addition, the remaining ones increase their size [57,58,59].

We could previously show, that 14 days after S100B immunization, more GABA-A receptor α3 and NMDA receptor 1 synapses could be detected, this is before a significant cell loss occurred. In contrast, gephyrin, as a marker for inhibitory post-synapses, was not altered [35]. As our analyses showed, gephyrin is also not affected after 28 days. Gephyrin itself acts as an anchor protein by binding GABA-A receptors and glycine to the post-synaptic skeleton [60,61]. In the brain tissue of patients with Alzheimer’s disease, an accumulation of gephyrin in co-localization with β-amyloid plaques was detected. Since gephyrin is involved in synaptic organization, the authors concluded that synaptic dysfunction is an early event in Alzheimer’s disease [62]. In the EAG model, gephyrin itself could not be found to be altered at different points in time, suggesting a minor role in the pathogenesis. However, the precise function of synaptic alterations should be investigated further.

Over the last years, the contribution of HSPs to glaucomatous damage has been widely discussed [63]. HSPs can serve as antigen-presenters, but they can also be recognized as antigens, and they are, therefore, connected to the immune response [64,65]. With our mass spectrometric analyses, we could observe a down-regulation of two peptide sequences of HSP60 in retinae 14 days after immunization. Previously, a study by Wax et al. revealed elevated serum levels of HSP60 in NTG patients [66]. Further, post-mortem immunostaining in retinae and optic nerve heads in patients with and without elevated IOP showed a higher intensity of HSP60. HSP60 was observed predominantly in RGCs and photoreceptors [67]. In the aqueous humor of patients with primary open-angle glaucoma (POAG), elevated levels of HSP60 and HSP90 were found [68]. Guo et al. compared POAG and NTG patients and noted higher serum antibody titers only in NTG subjects [69]. In contrast, a case-control study conducted in Poland could not determine an increase of serum HSP60 levels in NTG and POAG patients compared to control subjects [70].

As mentioned in the introduction, the EAG model is based on the finding of autoantibodies in glaucoma patients. When applying HSP60 systemically in rats, a loss of RGCs, predominantly in the central part of the retina, was observed after 28 days [71]. Contrarily, in rats from our study, that were immunized with the glial protein S100B, a down-regulation of HSP60 was detected by mass spectrometry. Similar results were noted after an intravitreal S100B injection. Here, mass spectrometry data also revealed a down-regulation of HSP60 when comparing S100B to PBS-injected eyes [72]. It is possible that S100B itself is responsible for the observed down-regulation. For instance, S100B can bind to RAGE. In a diabetic mouse model, the advanced glycation end product (AGE)/RAGE axis causes mitochondrial dysfunction in pancreatic islet cells and a down-regulation of HSP60 [73]. It is known that HSP60 down-regulation can lead to morphological changes, deficient ATP syntheses, inhibitions in cell proliferation, and decreases in mitochondrial membrane potential [74,75]. The results of the different studies indicate that both too much and too little HSP60 could contribute to cell damage.

A further interesting protein we found up-regulated after S100B immunization is α2-macroglobulin, a plasma acute-phase protein. It can bind to various ligands including cytokines [76,77], growth factors [78,79,80,81], and misfolded proteins [82,83,84,85]. For instance, α2-macroglobulin can bind to pro-inflammatory mediators, such as tumor necrosis factor α (TNFα), interleukin (IL)-1β, or IL-6 [86,87,88], leading to the assumption that it has an important role in controlling inflammatory mechanisms. Soluble α2-macroglobulin was noted in the aqueous humor of glaucoma patients as well as in a rat ocular hypertension (OHT) model [89]. Further, in OHT models (cauterization and saline injection), an up-regulation of α2-macroglobulin in retinae could be detected. These changes were long-lasting and continued even after pharmacological normalization of the IOP. In addition, the up-regulation occurred mainly in glia cells [90]. In our study, α2-macroglobulin was up-regulated in evaluations using mass spectrometry and immunohistology 7 and 14 days after S100B immunization. Hence, this protein might also play a role in RGC death, as proposed by Shi et al. [90]. In line with this, the neutralization of α2-macroglobulin in the vitreous and the inhibition of its function in the retina protected RGCs from glaucomatous damage [90,91,92].

Another acute-phase protein we found up-regulated after S100B immunization is serotransferrin. It is an iron-binding blood plasma protein that controls the levels of free iron in biological fluids [93]. Iron disbalance is known to increase oxidative stress and can, therefore, play a role in the pathogenesis of neurodegenerative diseases. For example, an increase of transferrin mRNA levels was observed in the temporal and frontal cortices of patients with Alzheimer’s disease [94]. Studies also located a link between elevated serotransferrin levels and glaucoma. In a proteomic analyses of human donor retinae from glaucoma patients, the authors revealed an up-regulation of serotransferrin [95]. Further, in serum samples of POAG patients, a higher concentration of serotransferrin was measured [96]. Hence, in combination with the results of elevated α2-macroglobulin levels, acute-phase proteins appear to play a crucial role in the pathogenesis of glaucoma and should be explored in more detail in further studies.

Interestingly, mass spectrometry of the probes revealed a regulation of certain proteins after S100B immunization, which are not directly linked to glaucoma or RGCs, such as IMPG1 and IMPG2. IMPGs belong to the ECM, which is the non-cellular component of all organs and tissues. The ECM in the retina can be divided into two entities: the interphotoreceptor matrix surrounding the inner and outer segments of the photoreceptors and the retinal ECM that surrounds the other cells [97]. IMPG1 and IMPG2 are involved in the development and maintenance of photoreceptors [98,99,100,101]. In the current study, IMPG1 was up-regulated in retinae at 7 and IMPG2 at 14 days after S100B immunization. Previously, an enhanced immunostaining of the ECM proteins Tenascin-C and phosphacan/RPTPβ/ζ was identified in S100B optic nerves after 7 days [41]. Tenascin-C showed enhanced immunoreactivity in the optic nerve after ischemia-reperfusion injury [102], while a knockout of Tenascin-C could protect the retinal function and rod-photoreceptor cells from ischemic damage [103].

Another protein found up-regulated 7 days after S100B immunization is myosin-10. It is an actin-based motor protein that participates in many essential intracellular processes, such as phagocytosis, cell migration, or filopodia formation [104]. In glaucomatous trabecular meshwork cells, myosin-10 distribution was disrupted [105]. In addition, myosin-10 might also play a role in focal ECM degradation in trabecular meshwork cells [106]. It is also known that myosin-10 binds to the NPxY motifs in the β-integrin cytoplasmic tail [107]. As a result of different integrin compositions, the ECM environment influences the regeneration and survival of adult RGC subtypes [108]. Hence, the remodeling of ECM proteins seems to contribute to retinal damage and a better understanding can provide new tools for diagnostic or therapeutic approaches in the future.

The observed results underline the multifactorial entity of the glaucoma disease. Immunological factors, especially, might serve as objective markers for diagnosis as well as disease progression in the future. Of course, these markers need to be further explored and validated in subsequent studies. Further, new therapeutic strategies could be developed based on the modulation and/or inhibition of immune system components. This could be used as adjuvant treatments in patients in addition to IOP-lowering drugs. However, new targets need to be explored first in (animal) models.

A limitation of our study is the fact that we performed proteomic analyses only at points in time where no RGC loss occurred. Although we specifically aimed to identify proteins early on in glaucoma, which might lead to cell death, it would also be interesting to perform those experiments at subsequent points in time after immunization. This would provide additional information about the changes of the retinal proteome in more chronic and advanced stages of glaucoma disease.

## 5. Conclusions

In our study, we identified 43 proteins which were significantly altered in S100B retinae after 7 days, and 67 altered proteins at 14 days. These shifts precede the loss of RGCs in S100B-immunized rats. Interestingly, serval regulated proteins were associated with the immune system, including beta-crystallin B1 and B3 or HSP60. Our observations provide comprehensive proteomic data to strengthen the pathophysiological impact of S100B and the involvement of the immune system in glaucoma. They can hopefully provide new hints towards disease markers or novel therapeutic approaches in the future.

## Figures and Tables

**Table 1:** The top 20 differential proteins 7 days after S100B immunization. Proteins quantified in ≥80% of the S100B and control groups are ranked based on the lowest *p*-value (≤0.02).

7 Days after S100B Immunization					
Statistical Rank	Uniprot ID	Protein Name	Qvalue (FDR) ≤0.01	*p*-Value ≤0.02	Ratio ≥30%
1	Q7TNY6	Golgi resident protein GCP60	1.38 × 10^−3^	0.002	48%
2	O35814	Stress-induced-phosphoprotein 1	1.15 × 10^−5^	0.002	48%
3	P25809	Creatine kinase U-type, mitochondrial	2.07 × 10^−7^	0.004	35%
4	P02770	Albumin	1.85 × 10^−10^	0.005	33%
5	P56574	Isocitrate dehydrogenase [NADP], mitochondrial	7.62 × 10^−4^	0.006	53%
6	P63004	Platelet-activating factor acetylhydrolase IB subunit alpha	3.21 × 10^−5^	0.007	45%
7	P12785	Fatty acid synthase	2.00 × 10^−6^	0.007	57%
8	P49432	Pyruvate dehydrogenase e1 component subunit beta, mitochondrial	3.79 × 10^−6^	0.010	108%
9	P12346	Serotransferrin	2.08 × 10^−8^	0.011	33%
10	Q63151	Long-chain-fatty-acid--CoA ligase 3	7.13 × 10^−4^	0.012	45%
11	P14841	Cystatin-C	3.09 × 10^−4^	0.012	61%
12	Q63598	Plastin-3	1.39 × 10^−3^	0.012	85%
13	Q9JLT0	Myosin-10	1.75 × 10^−4^	0.013	43 %
14	Q924S5	Lon protease homolog, mitochondrial	4.22 × 10^−5^	0.015	40%
15	O70351	3-hydroxyacyl-CoA dehydrogenase type-2	9.91 × 10^−4^	0.016	85%
16	Q8K1P7	Transcription activator BRG1	3.13 × 10^−9^	0.018	55%
17	P15146	Microtubule-associated protein 2	3.03 × 10^−5^	0.019	58%
18	Q5PPM7	Rod outer segment membrane protein 1	2.64 × 10^−9^	0.021	51%
19	P21531	60S ribosomal protein L3	6.73 × 10^−5^	0.021	31%
20	Q5XI73	Rho GDP-dissociation inhibitor 1	5.02 × 10^−7^	0.021	77%

**Table 2:** The top 20 differential proteins 14 days after S100B immunization. Proteins that are quantified in ≥80% of the S100B and control groups are ranked based on the lowest *p*-value (≤0.02).

14 Days after S100B Immunization					
Statistical Rank	Uniprot ID	Protein Name	Qvalue (FDR) ≤0.01	*p*-Value ≤0.02	Ratio ≥30%
1	Q5XI31	GPI transamidase component PIG-S	3.05 × 10^−3^	0.003	36%
2	P11506	Plasma membrane calcium-transporting ATPase 2	1.37 × 10^−8^	0.003	54%
3	P41542	General vesicular transport factor p115	1.81 × 10^−4^	0.003	46%
4	P63039	60 kDa heat shock protein, mitochondrial	4.80 × 10^−11^	0.004	45%
5	P21670	Proteasome subunit alpha type-4	2.04 × 10^−7^	0.005	69%
6	Q9JK11	Reticulon-4	3.50 × 10^−9^	0.010	37%
7	Q62927	cGMP-gated cation channel alpha-1	5.20 × 10^−6^	0.011	30%
8	Q9ePH8	Polyadenylate-binding protein 1	1.99 × 10^−7^	0.012	59%
9	Q6IMY8	Heterogeneous nuclear ribonucleoprotein U	4.57 × 10^−9^	0.012	109%
10	P14881	Beta-crystallin A3	2.14 × 10^−10^	0.013	298%
11	D3ZLZ7	Inosine-5’-monophosphate dehydrogenase 1	5.11 × 10^−10^	0.015	32%
12	O35476	Medium-wave-sensitive opsin 1	1.18 × 10^−4^	0.016	152%
13	P63012	Ras-related protein Rab-3A	1.34 × 10^−8^	0.016	41%
14	P84083	ADP-ribosylation factor 5	9.07 × 10^−8^	0.017	37%
15	P06761	endoplasmic reticulum chaperone BiP	8.01 × 10^−9^	0.018	71%
16	P02770	Albumin	3.75 × 10^−11^	0.018	45%
17	D3ZHV2	Microtubule-actin cross-linking factor 1	2.31 × 10^−4^	0.018	37%
18	P62752	60S ribosomal protein L23a	4.00 × 10^−4^	0.019	42%
19	Q68FX0	Isocitrate dehydrogenase [NAD] subunit beta, mitochondrial	4.00 × 10^−7^	0.020	32%
20	P50475	Alanine--tRNA ligase, cytoplasmic	5.49 × 10^−8^	0.020	78%

## Data Availability

Mass spectrometry data set of the study samples has been uploaded to ProteomeXchange with the identifier PXD023995.

## Supplementary Materials

The following are available online at https://www.mdpi.com/article/10.3390/biology11010016/s1, Table S1: Primary and secondary antibodies for immunohistology. Table S2: A total of 1287 proteins identified with at least one unique peptide in at least 80% of the control as well as the S100B samples at 7 days. Table S3: A total of 1384 proteins identified with least one unique peptide and d in at least 80% of each study group at 14 days. Figure S1: Pathway analysis of rat retina-specific spectral library proteins. Figure S2: Pathway analysis of retinal proteins 7 (A, B*) and 14 days (C, D*) after S100B immunization. Figure S3: STRING protein–protein interaction network of significant proteins 7 days after S100B immunization. Figure S4: STRING protein–protein interaction network of significant proteins 14 days after S100B immunization.
