# Supplementary material for: Proteomic Analysis of Retinal Tissue in an S100B Autoimmune Glaucoma Model
Source: Biology (Basel). 2021 Dec 23;11(1):16. doi: 10.3390/biology11010016 (PMC8773367; doi:10.3390/biology11010016)
Supplement: Supplementary file 1 [file biology-11-00016-s001.zip › Supplementary figures.pdf]

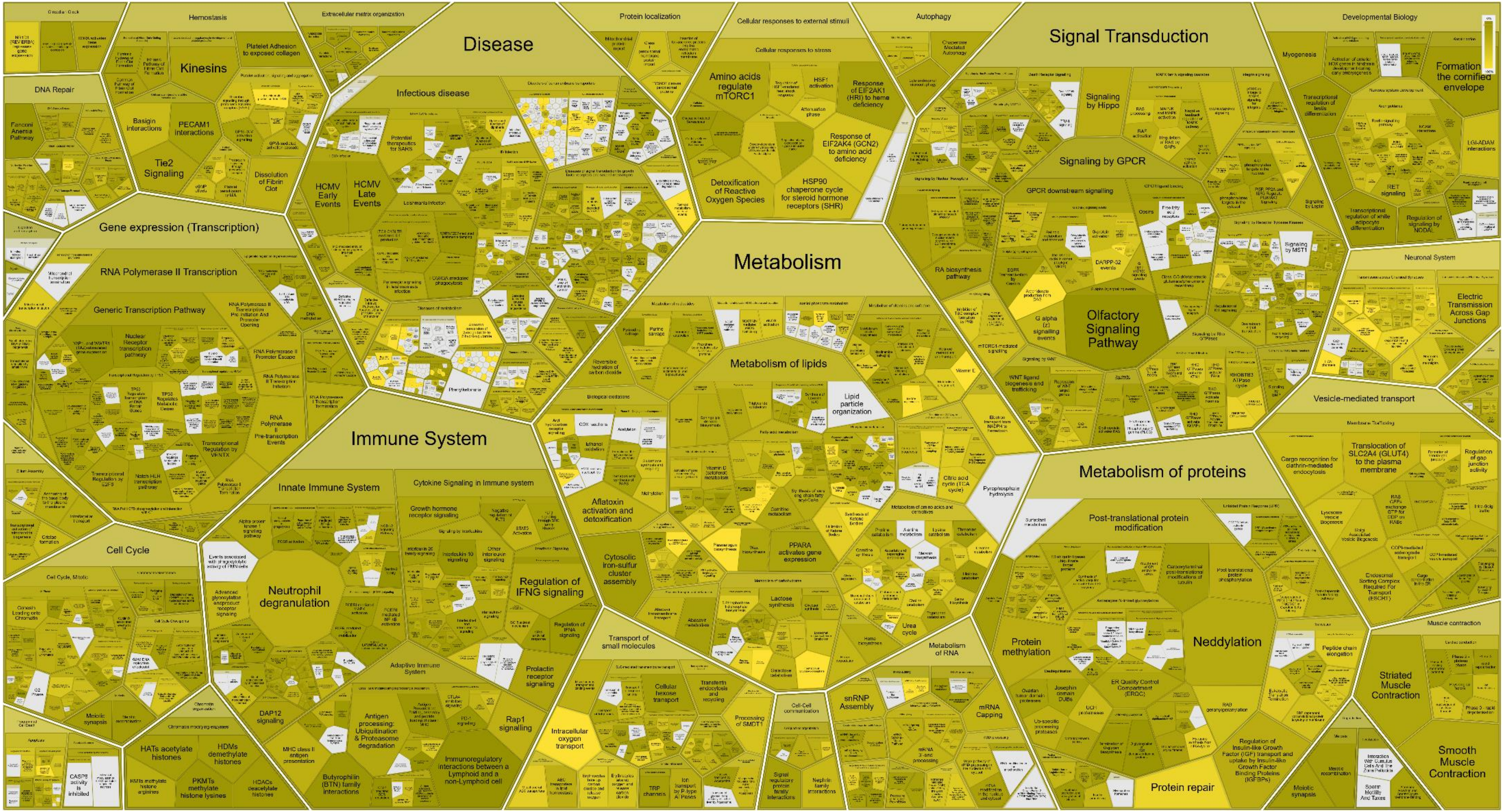

Figure S1: Pathway analysis of rat retina-specific spectral library proteins. Detailed pathway overview of 2725 spectral library proteins (out of 4689 proteins), where 1988 pathways were hit by at least one of them. Each cell represents a biological pathway. From copper to yellow with increasing overrepresentation.

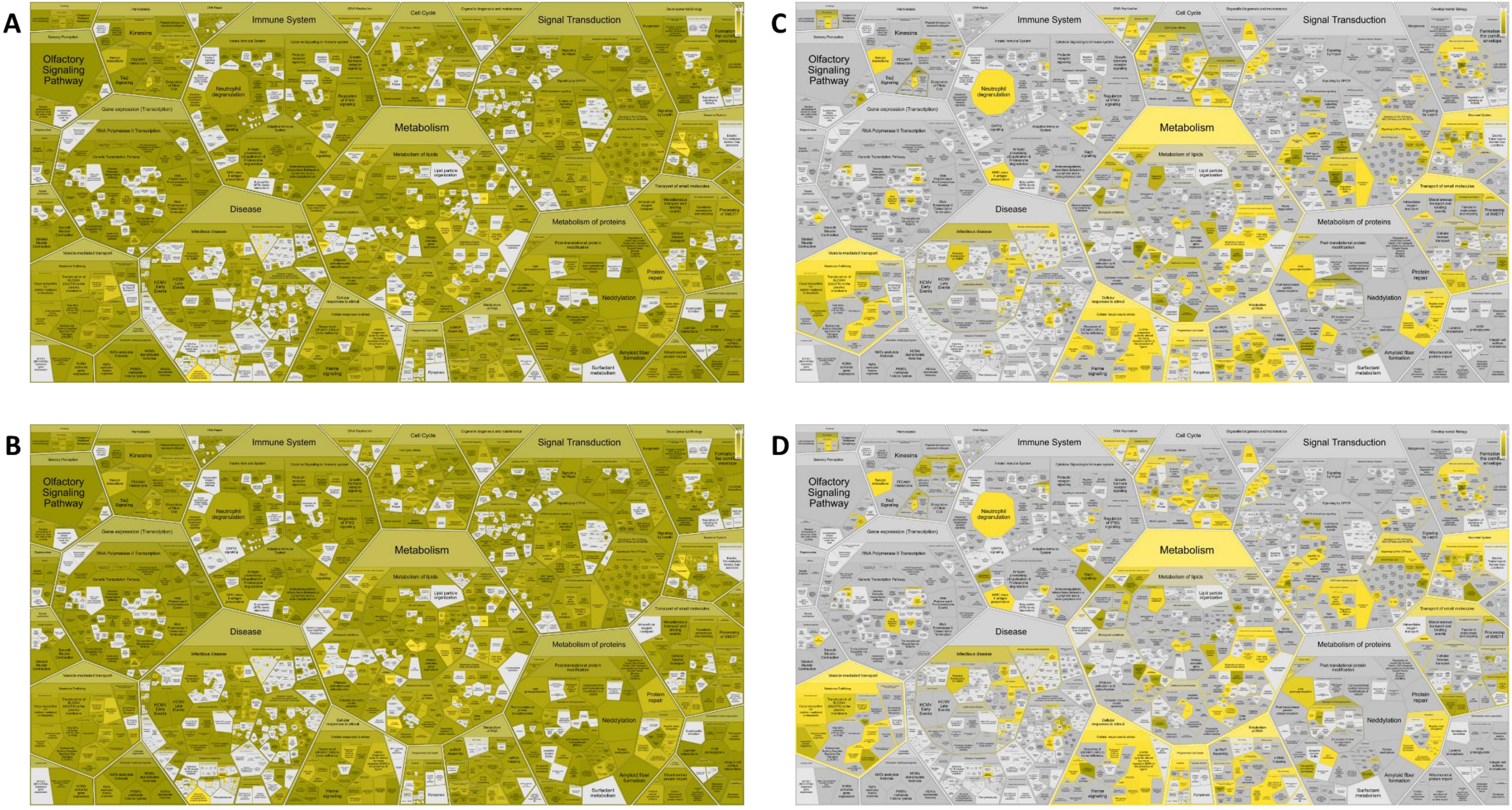

Figure S2: Pathway analysis of retinal proteins 7 (A, B) and 14 days (C, D) after S100B immunization. **A)** Pathway overview of 1641 biological pathways associated with 1225 proteins (out of 1744 proteins). **B)** Overrepresentation of significant pathways based on identified proteins 7 days after S100B immunization. **C)** Pathway overview of 1234 proteins (out of 1752 proteins), where 1637 pathways were hit by at least one of them. **D)** Overrepresentation of significant pathways based on identified proteins 14 days after S100B immunization. ⁺From copper to yellow with increasing overrepresentation. *From yellow to copper to a more significant p-value (Benjamini–Hochberg procedure).

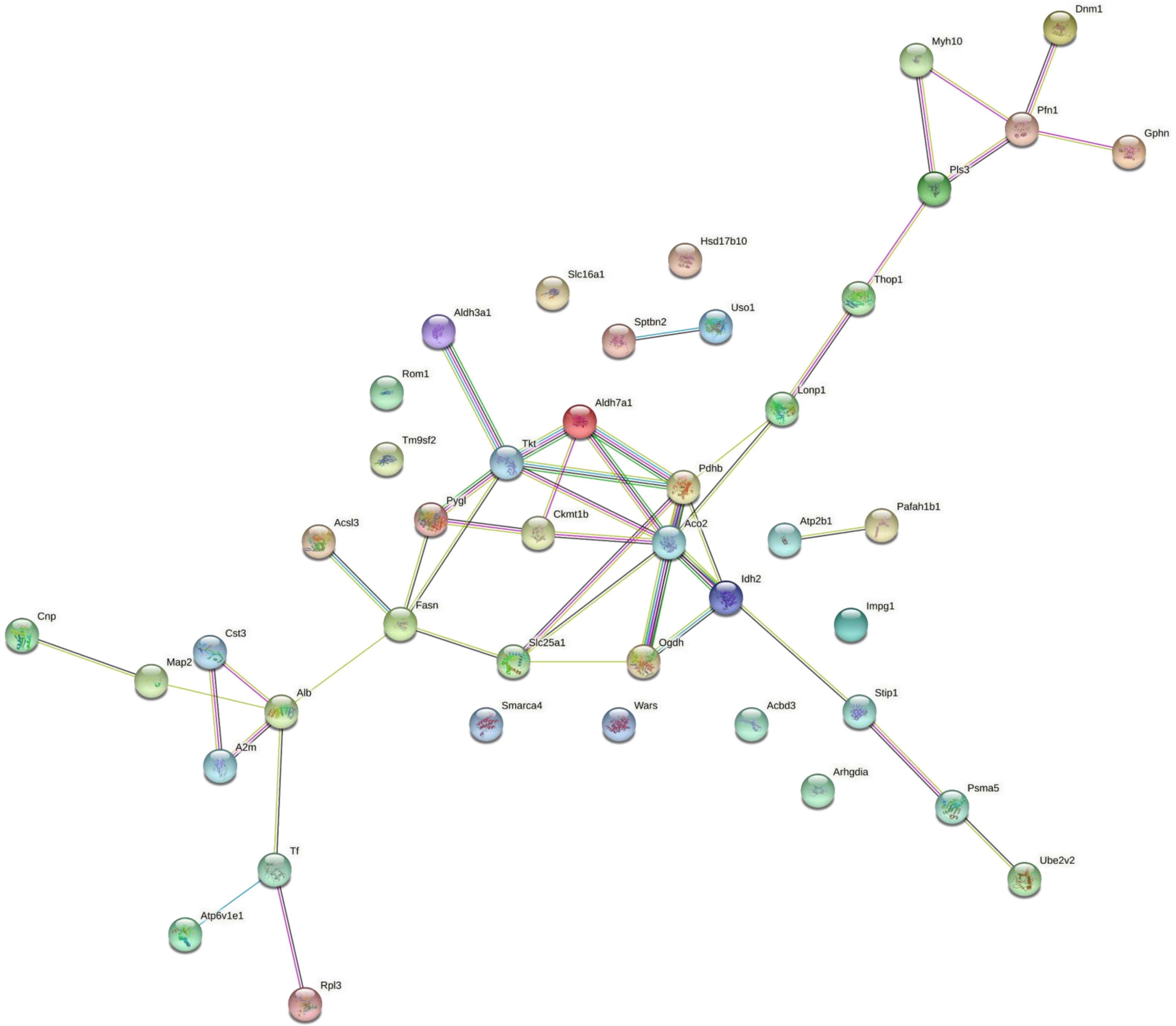

Figure S3: STRING protein-protein interaction network of significant proteins 7 days after S100B immunization.

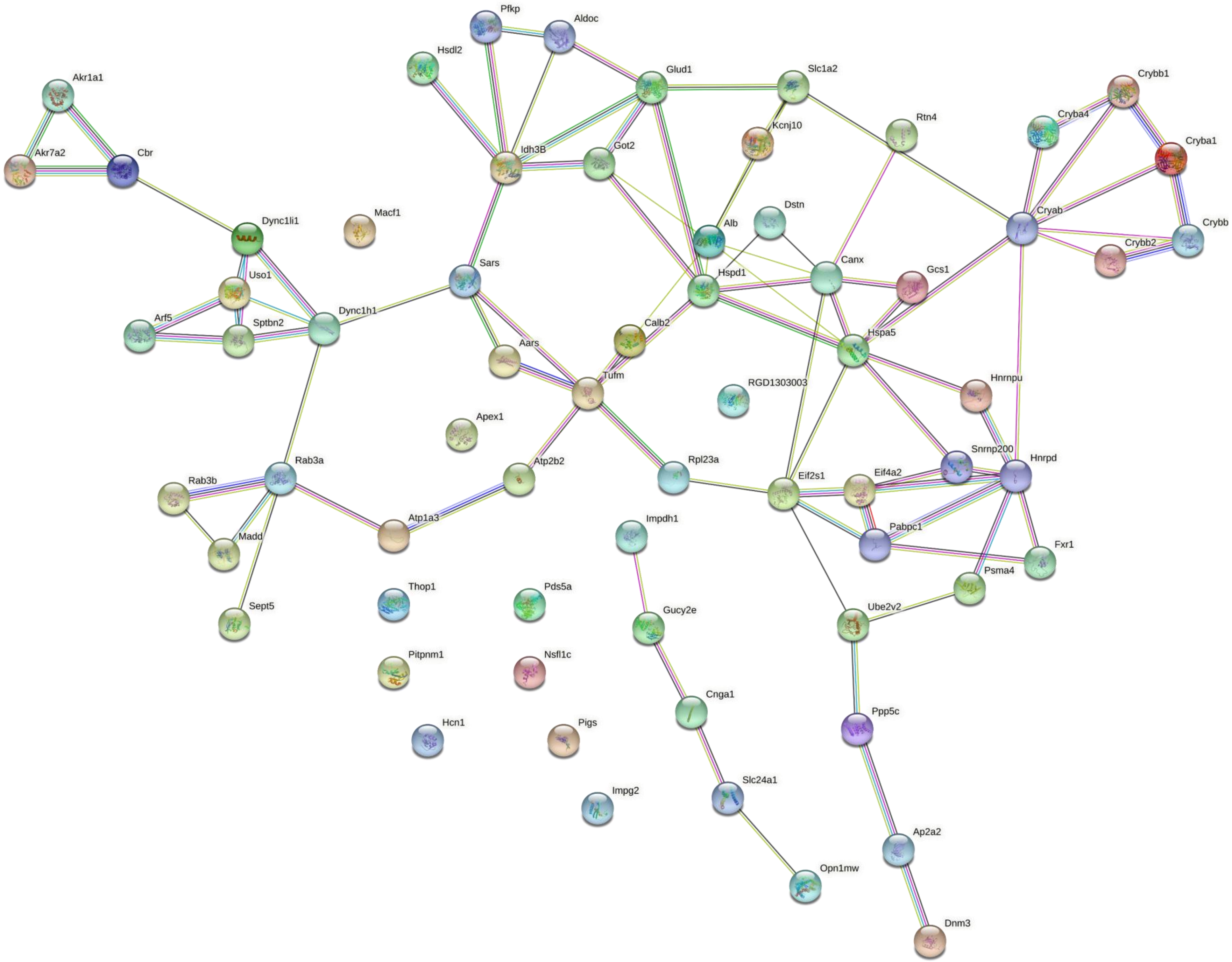

Figure S4: STRING protein-protein interaction network of significant proteins 14 days after S100B immunization.
