# Supplementary material for: Proteomic Analysis of Retinal Tissue in an S100B Autoimmune Glaucoma Model
Source: Biology (Basel). 2021 Dec 23;11(1):16. doi: 10.3390/biology11010016 (PMC8773367; doi:10.3390/biology11010016)
Supplement: Supplementary file 1 [file biology-11-00016-s001.zip › Table S1.pdf]

Table S1: Primary and secondary antibodies used for immunohistology.

Primary antibody	Company	Dilution	Secondary antibody	Company	Dilution
Anti-α2-macroglobulin	Thermo Fisher	1:100	Donkey anti-rabbit Alexa Fluor 555	Invitrogen	1:500
Anti-Calretinin	Millipore	1:2000	Donkey anti-goat Alexa Fluor 488	Dianova	1:500
Anti-GFAP	Millipore	1:700	Donkey anti-chicken Alexa Fluor 488	Jackson ImmunoResearch	1:500
Anti-HSP60	Sigma-Aldrich	1:100	Donkey anti-rabbit Alexa Fluor 555	Invitrogen	1:500
Anti-IMPG1	Novus Biological	1:100	Donkey anti-rabbit Alexa Fluor 555	Invitrogen	1:500
Anti-IMPG2	Sigma-Aldrich	1:100	Donkey anti-rabbit Alexa Fluor 555	Invitrogen	1:500
Anti-PLS3	Sigma-Aldrich	1:100	Donkey anti-rabbit Alexa Fluor 555	Invitrogen	1:500
Anti-RBPMS	Millipore	1:500	Donkey anti-rabbit Alexa Fluor 555	Invitrogen	1:500
